# *Stevia Rebaudiana* Bertoni, a Source of High-Potency Natural Sweetener—Biochemical and Genetic Characterization

**DOI:** 10.3390/molecules25040767

**Published:** 2020-02-11

**Authors:** Magdalena Dyduch-Siemińska, Agnieszka Najda, Jacek Gawroński, Sebastian Balant, Klaudia Świca, Agnieszka Żaba

**Affiliations:** 1 Department of Genetics and Horticultural Plant Breeding, Institute of Plant Genetics, Breeding and Biotechnology, University of Life Sciences in Lublin, Akademicka 15 Street, 20-950 Lublin, Poland; magdalena.dyduch@up.lublin.pl (M.D.-S.); jacek.gawronski@up.lublin.pl (J.G.); katedra.genetyki@up.lublin.pl (A.Ż.); 2 Department of Vegetable Crops and Medicinal Plants, University of Life Sciences, Akademicka 15 Street, 20-950 Lublin, Poland; sebastianbalant@o2.pl (S.B.); klauskoczek@poczta.onet.pl (K.Ś.)

**Keywords:** *Stevia rebaudiana* Bertoni, steviol glycosides, polyphenolics, phenolic acids, tannins, flavonoid, RAPD

## Abstract

*Stevia rebaudiana* is a natural sweetener herb that is increasingly used in herbal medicines in the food and cosmetics industries. Molecular methods can be combined with morphological techniques to identify stevia genotypes as a starting material to produce more reliable bioproducts. This study evaluated the level of the genetic and biochemical diversity in various stevia genotypes using HPLC (high performance liquid chromatography) analysis and random amplified polymorphic DNA (RAPD) markers. Stevia genotypes collected from different locations of the world showed clear variations at the biochemical and genetic level in Polish climate conditions. The influence of the genotypes on the content of steviol glycosides, antioxidants, phenols, flavonoids, and tannins was analyzed using phytochemical assays. Genotypes from Morocco, Poland, Egypt, and Nigeria can be defined as samples of higher quality compared to other genotypes analyzed in terms of the amount of steviol glycosides. Considering the rebaudioside A/stevioside ratio as a selection criterion, genotypes from Australia, China, India, and Pakistan should be considered to be valuable in terms of suitability for obtaining new varieties. The present results of RAPD marker analysis indicated differential banding pattern and considerable polymorphism among all ten stevia genotypes. Genotypes from Morocco, Egypt, Poland, Nigeria, China, and India, as genetically different, can be selected for further stevia breeding programs.

## 1. Introduction

There is a growing tendency for people to consume foods low in sugar and calories*. Stevia rebaudiana* Bertoni (*Asteraceae* family) is a natural sweetener herb [1]. Stevia leaves produce secondary metabolites (diterpene glycosides), which are about 300 times sweeter than sucrose [2]. The Japanese have performed over 40,000 clinical studies which demonstrated that steviol glycosides are safe when used as a sweetener. Possible medicinal applications have been often investigated using stevia extracts as intravenous infusions in rats to reveal its effect on glucose metabolism, diuresis, organ weights, endocrine function or anti-androgenic activity. Stevia extracts have also been shown to be likely beneficial as antioxidants and blood pressure and hypertension reducers [3]. All the above-mentioned properties of steviol glycosides allow their wide use in herbal medicines (tonics for diabetic patients), food industry (desserts, sauces, ketchups, drinks, bread, fruit juices, formulas for athletes) and cosmetics (mouthwashes and toothpastes, creams) [4]. The increasing demand for natural sweeteners have led farmers in many countries to grow stevia on a large-scale, and cultivation studies have also been carried out in different parts of the world, from Asia to the Americas [5]. Stevia plants grown in Europe are often undefined varieties, propagated mainly by conventional vegetative methods. They are characterized by high genetic diversity, which does not guarantee the production of steviol glycosides and other phytochemicals at a constant high level. Food additives and medicinal preparations based on such plants do not provide a uniform high standard. Therefore, the production of phytoproducts from this type of plants is not possible on a commercial scale. Hence a comprehensive description of stevia plants as the starting material to produce bioproducts at both the biochemical and molecular level is very important. Molecular methods could be combined with morphological techniques to identify stevia genotypes more reliably. Molecular markers are valuable tools in the characterization and evaluation of genetic diversity within and between cultivars and populations. It has been shown that different markers can reveal different classes of variation. This is correlated with the genome fraction surveyed by each type of marker, their genome distribution and DNA target range analyzed by each specific assay [6]. Molecular characterization helps determine species breeding behavior of, individual reproductive success and the presence of gene flow, alleles transfer within and between populations of the same or related species, and its consequences [7]. Random amplified polymorphic DNA (RAPD) is a convenient method to detect genetic diversity. RAPD has several advantages, such as simplicity of use, low cost, and use of small amounts of plant material. RAPD markers has been proven to be useful as genetic markers for cross-pollinated *Stevia* crops, in which high genetic diversity have been observed [3,6,8]. A great advantage of RAPD markers is that it is not necessary to know the sequence of a DNA template, whereas a short length of primers means that the markers are distributed throughout the genome in both coding and non-coding regions [9,10]. RAPD markers are highly polymorphic and allow the distinguishing of even very similar genotypes [11]. This technique can be combined with others, such as RFLP (restriction fragments length polymorphism), SSR (simple sequence repeats) or ISSR (inter simple sequence repeats) in order to obtain a more accurate and specific markers [10,11]. From RAPD markers, after sequencing their products, more specific SCAR (sequence characterized amplified region) markers can be obtained [12]. Recognition of genetic and biochemical diversity is the basis for starting breeding programs and is essential for the selection of plant genotypes. In this study, we evaluated the level of genetic and biochemical diversity in various genotypes of stevia from different regions of the world using HPLC analysis and RAPD markers to establish a baseline to assist future breeding programs of this species.

## 2. Results and Discussion

### 2.1. Chemical Analysis

The sum of steviol glycosides in the studied group of genotypes ranged from 5.22–12.97%. Literature data indicate a varied content of steviol glycosides depending on the growth conditions and cultivation methods, the amount of which may range from 2% to 20% fresh leaf weight [13,14]. According to Mishra et al. [15] stevioside is the main sweetener in the leaves of *Stevia rebaudiana* plants (from 5–15% dry matter); the following are rebaudioside A (2–6% dry matter), rebaudioside C (1–2% dry matter) and dulcoside A (0.4–0.7% dry matter). Stevioside content in the studied group of genotypes ranged from 2.68% in the genotype from India to 8.09% in the genotype from Pakistan (Table 1). The values for stevioside obtained in this study were higher than those reported by Parris et al. [16] and Vouillamoz et al. [17]. Raina et al. [18] analyzed stevia leaves from India and showed the presence of stevioside in the range from 4.25% to 7.32%, and rebaudioside A in the range from 2.01% to 4.13%, which was confirmed in the present study. Higher values were obtained by Pereira et al. [19], who reported that stevioside content in stevia leaves ranged from 6.98% to 12.16%. A comparison of rebaudioside A content in the studied group of genotypes showed the lowest content (1.17%) of this compound in the genotype from Malaysia, and the highest (4.48%) in the genotype from Australia. The determined content of rebaudioside A was lower than that reported by Parris et al. [16], Pereira et al., [19] and Vouillamoz et al. [17]. In turn, Gardana et al. [20], Goyal et al. [4], Atteh et al. [21], Jaworska et al. [22] and Khiraoui et al. [14] obtained lower rebaudioside A content in the raw material.

According to Makapugay et al. [23] and Geuns [24], rebaudioside B, D, E and F constituted trace components. In our research, we demonstrated that rebaudioside C and D were in fact components with the lowest concentration in the analyzed raw material. We have proved that genotypes originating in Malaysia and Paraguay contained the lowest amounts of rebaudioside C and D, and those derived from China and Australia the highest. 

This result has confirmed that, as with other secondary metabolites, stevia glycoside profile is subject to significant variations depending on the geographical area, the state of plant maturity, environmental conditions, harvest date and processing method. Since changes in the composition of the plant matrix can affect its sweet taste, as well as modify its therapeutic and toxic properties, it is necessary to control the quality of the raw material to ensure the effectiveness and safety of the use of this species in food and medicine. At the same time, we observed in our studies a higher content of rebaudioside D in genotypes in which we found the lowest stevioside content. It is interesting that in the analyzed genotypes, we did not identify steviol and rebaudioside B, E and F. Two steviols present in plant tissue, stevioside and rebaudioside A, are largely responsible for the sweet flavor of stevia leaves [8]. It should be considered that the total content of stevioside and rebaudioside A indicates the quality of the raw material, and the results listed in Table 1 show differences in the content of these compounds, depending on seed origin. Thus, genotypes from Morocco, Poland, Egypt, and Nigeria had a high concentration of compounds with a high sweetness index and low toxicity. Therefore, genotypes from Morocco, Poland, Egypt and Nigeria can be defined as samples of higher quality compared to other genotypes analyzed for the amount of steviol glycosides. Stevia breeding programs should aim at improving the total glycoside content and rebaudioside A/stevioside ratio with a higher leaf yield [25,26]. The native rebaudioside A/stevioside ratio in stevia leaves is usually about 0.5 or less [26]. Assuming the rebaudioside A/stevioside ratio above 1 as a selection criterion, similarly as Kaplan and Turgut [2], genotypes from Australia (genotype 9), China (genotype 1), India (genotype 3) and Pakistan (genotype 8) should be considered valuable in terms of suitability for obtaining new varieties. 

Total phenol content expressed in mg·g^−1^ dry weight (DW) of tested samples in gallic acid equivalent (GAE) is given in Table 2. Significant differences (*p* < 0.05) in the total phenolic content between the tested genotypes were shown. Genotypes from Paraguay, Malaysia and India had the highest total polyphenolics (TP). In addition, the content of phenolic acids and tannins in these genotypes was the highest, but it was shown that the amount of flavonoids (mg catechin equivalent/g stevia) in the leaves of these genotypes was the lowest. It is significant that in relation to total flavonoids, the highest values (12.99 to 12.38 mg·g^−1^ DW) were obtained for genotypes in which the lowest TP, TPA and total tannins (TT) contents were determined. At the same time, leaf extracts of these genotypes showed the greatest tolerance to DPPH• free radicals. The results obtained with respect to the total level of phenols, flavonoids and antioxidant activity were consistent with those reported by Periche et al. [27] on stevia leaves dried in conventional conditions. However, no studies have been found on the comparison of genotypes in terms of phenolic acid content.

### 2.2. Molecular Analysis

It is necessary to screen and identify different genotypes for various applications, including secondary metabolite production or plant improvement by breeding. Several studies have shown that RAPD analysis can be successfully applied to identify variations in stevia genome. Thiyagarajan and Venkatachalam [8] reported genomic DNA polymorphisms and phytochemical variation of *Stevia rebaudiana* (Bertoni) using RAPD-PCR of three *S. rebaudiana* accessions (L1 to L3). Gupta et al. [25], analyzed the variation at morphological and molecular (RAPD) levels and performed chemodiversity analysis using LC-MS among seven ecotypes collected from different states of India. Genetic variability analysis in *Stevia rebaudiana* collected from different locations in the world was carried out in this study using RAPD markers. *Stevia rebaudiana* Bert. genotypes were analyzed using 40 RAPD primers (Sigma–Aldrich), of which 10 produced a high number of polymorphic and repeatable fragments. Nucleotide sequences of these primers are shown in Table 3.

**Figure 1:**
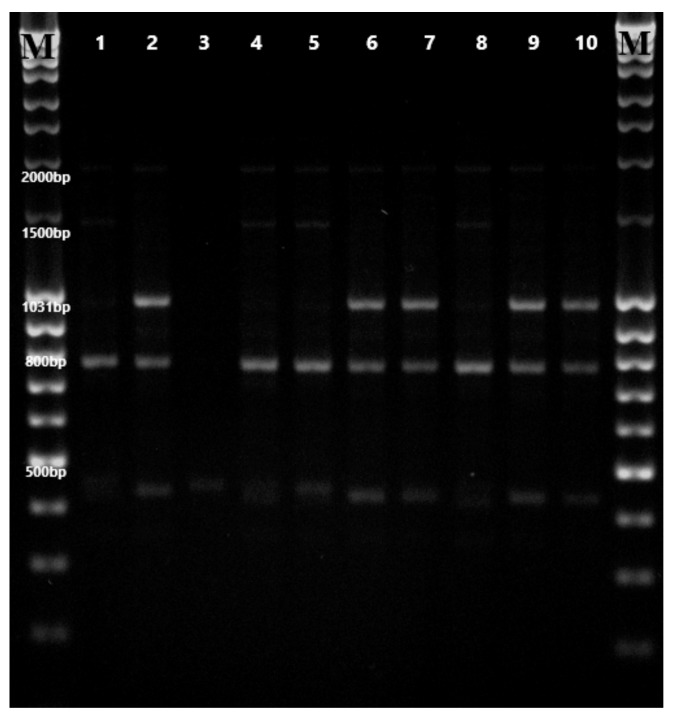
RAPD fingerprints of ten stevia genotypes using primer RAPD 2. M—standard of DNA fragment size GeneRulerTM DNA Ladder Mix (100–10,000 bp).

Genotype amplification products with the use of these 10 primers yielded a total of 79 scorable bands. The size of the amplification products ranged from 400–2200 bp. Selected primers produced 57 polymorphic fragments. The analyzed DNA fragments showed variation in the presence of polymorphisms. The product amplified by primer 9 had the highest polymorphism (87.5%), while fragment amplified by primer 10 exhibited the lowest polymorphism (60%). A similar proportion of polymorphism (67.24–92.40%) was observed by Chester et al. [28], who analyzed 11 genotypes using 10 RAPD primers. The average polymorphism value for this study (72%) was similar to the results obtained by Thiyagarajan and Venkatachalam [8]—81%—and Gupta et al. [25]—67%. Ten RAPD primers amplified a different number of monomorphic and specific products. The highest number of monomorphic bands was produced by primers 2 and 3. Primers 2, 5, 7, 9 did not amplify any specific products. An example of RAPD fingerprints of ten stevia genotypes using primer 2 is presented in Figure 1. The Rp value of ten RAPD primers ranged from 1.2 to 6.4 (Table 3). In the current study, RAPD primers with high resolving power values were able to clearly separate most of the analyzed genotypes. Variations in genetic structure within a species are usually associated with geographic range, mode of reproduction, mating system, seed dispersal and fecundity [25]. The genetic diversity detected in the present study may be caused by some or all these factors. This is consistent with the study of Thiyagarajan and Venkatachalam [8] and Gupta et al. [25], who reported the presence of genetic variation in *S. rebaudiana* accessions from different regions of India.

The similarity coefficients between genotypes were calculated based on 10 RAPD primers, (Table 4), and they varied from 0.50 to 0.92 (mean—0.68).

**Figure 2:**
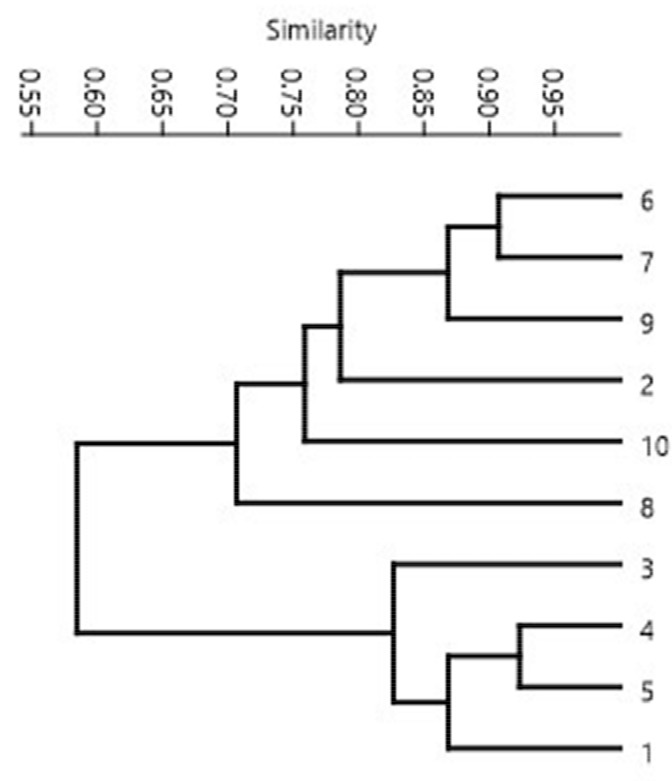
UPGMA dendrogram of 10 stevia genotypes based on RAPD markers.

The lowest value of the genetic similarity was detected between genotypes 1 and 9. Cluster analysis based on the UPGMA (Unweighted Pair-Group Method Using Arithmetic) method allowed Chester et al. [28] to reveal genetic diversity between 11 genotypes from different regions of India. In our research, UPGMA analysis was also used to group the genotypes. Cluster identification on the dendrogram was performed at genetic similarity level of 0.68, which was the mean value for the RAPD marker system. The analysis of the dendrogram (Figure 2) indicated the presence of two main clusters. The first cluster contained six genotypes (2, 6, 7, 8, 9, 10). The remaining 4 genotypes formed a separate cluster with a similarity range of 0.83-0.92. Three of the four genotypes grouped together in this cluster were from the Asian region, which suggested that they were a closely related germplasm, which may be due to being cultivated at the closest latitude. Among the genotypes grouped in the first cluster, the highest similarity was found between genotypes 6 and 7 originating in the North African region. 

**Figure 3:**
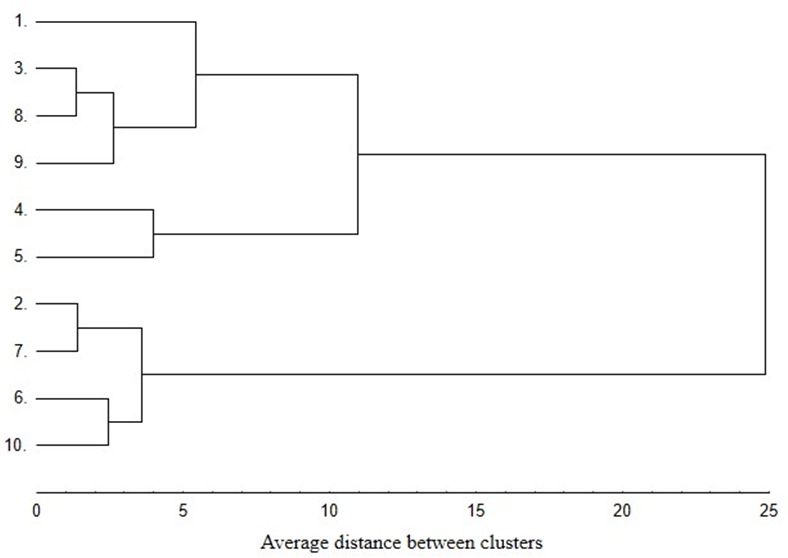
Dendrogram estimating distance between 10 stevia genotypes based on steviol glycosides (% DW) and polyphenols (mg·g^−1^ DW) concentration.

In the case of the dendrogram generated for phenotypic traits (Figure 3), a slightly different clustering pattern was obtained for the analyzed group of genotypes. Considering the concentration of steviol glycosides (% DW) and polyphenols (mg·g^−1^ DW), it was also possible to distinguish two main clusters. The first contained two subclasters. One of them included only two stevia genotypes (4, 5), which were grouped together also in the dendrogram based on RAPD markers. Apart from that, all the values of the traits analyzed in Table 1 and Table 2 were very similar for these genotypes. The greatest similarity within the second subcluster occurred between the genotypes originating in India and Pakistan, to which accessions from Australia and China were subsequently added. These genotypes were characterized by a high rebaudioside A/stevioside ratio and low DPPH• value. The second cluster contained other 4 genotypes, i.e., 2, 7, 6, 10, which had a low rebaudioside A/stevioside ratio, high total steviol glycoside content and high DPPH• value. 

The heritability coefficient (h^2^) is one of the parameters enabling selection and breeding programs aimed at obtaining higher yields or improving quality traits of stevia. The presence of a significant heritability coefficient (h^2^) for three economically important traits, i.e., leaf yield, leaf/stem ratio and stevioside content (62.1, 78.8 and 76.6, respectively) has suggested that genetic improvement of stevia is possible [27,29]. Given the position of the genotypes on the dendrogram (Figure 2) in relation to the total steviol glycoside contents—rebaudioside A + stevioside (Table 1)—it was found that genotypes 6, 7, 2 and 10, containing the highest content of these compounds, were grouped in one clade. In turn, genotypes 1 and 3, with a high value of the rebaudioside A/stevioside ratio, were also grouped together, but in a separate clade with respect to the previous group. Therefore, the low genetic similarity between these groups creates an opportunity to use combination breeding methods to obtain genotypes characterized by a high content of steviol glycosides and a higher rebaudioside A/stevioside ratio. Nevertheless, work on combination breeding of Stevia rebaudiana is currently scarce and studies conducted two decades ago were based solely on phenotypic selection. Selection carried out by Kalpan and Turgut [2] in populations obtained from open pollination for high rebaudioside A content and high rebaudioside A/stevioside ratio indicated the possibility of obtaining such genotypes. Therefore, our research has shown that the inclusion of genotypes selected on the basis of RAPD markers 6, 7, 2, 10, 1 and 3 in the crossbreeding program should create genetic variation and as a consequence, may enable the selection of the most desirable accessions, which will be more useful for consumers and bioindustry.

## 3. Materials and Methods 

### 3.1. Plant Material

The raw material for research was obtained from agrotechnical experience of the Department of Vegetable and Medicinal Plants. Stevia seeds were obtained from 10 institutions and companies involved in breeding new varieties of stevia (Table 5). Sowing of seeds was carried out in a heated greenhouse in the second decade of January 2018 to flowerpots filled with horticultural substrate. Plants were planted in permanent place in the first decade of May. Samples of healthy leaves from 10 randomly selected plants were collected after 120 days of vegetation in the field, i.e., on August 28, 2018, grown at the Experimental Farm of the Department of Vegetable and Medicinal Plants of the University of Life Sciences in Lublin (51°14′53″N, 2°34′13″E). Immediately after harvesting, the leaves were frozen in liquid nitrogen and stored at −20 °C until analysis. Raw material samples were ground before analysis. The sample from each sample is stored in the Department of Vegetable Plants and Medicinal Plants of the University of Life Sciences in Lublin, Poland, under the numbers ST1/2018/340, ST2/2018/341, ST2/2018/342, ST4/2018/343, ST5/2018/344, ST6/2018/345, ST7/2018/346, ST8/2018/347, ST9/2018/348, ST10/2018/349.

### 3.2. Phytochemical Assays

#### 3.2.1. Sample Preparation and Extraction

10 g of air-dried and crushed leaves were extracted in a Soxhlet apparatus in 150 mL of methanol for 8 h. Then the extract was evaporated to dryness and diluted with 10 mL of acetonitrile. The diluted samples were centrifuged (5 min, 21,000× *g*, Universal 32, Hettich, Germany), filtered through a 0.2 μm membrane syringe filter (Costar X, Corning Inc., Salt Lake City, UT, USA) and transferred to a glass vial.

#### 3.2.2. Fractionation of Steviol Glycosides Compounds using HPLC 

Steviol glycosides were analyzed using a LaChrom-Merck HPLC system equipped with a DAD (Diode Aral Detection) diode detector (L-7450), binary pump (L-7100), degasser (L-7612), thermostat (L-7360), Rheodyne injector and LiChrospher 100 steel column RP C18 (250 mm × 4 mm) filled with stationary phase (dp = 5 μM). UV detection was carried out at 210 nm at 28 °C and a flow rate of 0.750 mL/min. Separation was achieved in a gradient mode water: acetonitrile (A: B), 0–4 min 100–70% B, then 5–10 min 70–35% B, then return to B in 5 min. To increase separation, 0.005% formic acid was added to both mobile phases. Stevioside, rebaudioside A, rebaudioside B, rebaudioside C, rebaudioside D, steviol were monitored. The results were compared with those obtained for pure standards.

#### 3.2.3. Quantification of Phenolics

*Total polyphenolics* (TP) were determined in dry extracts with the Folin–Ciocalteau procedure [30]. To prepare the calibration curve, 0.1 mL aliquots of 0.037, 0.072, 0.108, 0.144, and 0.108 mg/mL of ethanolic gallic acid solutions were mixed with 7.9 mL of H_2_O, 0.5 of Folin–Ciocalteau reagent, and 1.5 mL of 20% sodium carbonate. The absorption was read after 2 h at a temperature of 20 °C at 765 nm and the calibration curve was constructed. 0.1 mL of the methanolic plant extract (1 mg/mL) was mixed with the same reagents as described above, and after 2 h the absorption was measured for the determination of plant phenolics. The content of total phenolic compounds in the investigated plant methanolic extracts was expressed as mg of GAE per 1 g of the DW.

Estimation of *total phenolic acids* (TPC) was carried out according to the Arnov method [31]. One milliliter of the sample was mixed with 5 mL of distilled water, 1 mL of 0.5 M HC1, 1 mL of Arnov reagent, and 1 mL of NaOH. Subsequently, the total acid content was expressed as caffeic acid equivalent (CAE) in mg·g^−1^ DW.

*Total flavonoid* (TF) content was measured by means of the aluminum chloride colorimetric assay [32]. A 1L aliquot of 0.037, 0.074, 0.112, 0.149, and 0.186 mg/mL of methanolic rutin solutions or methanolic plant extracts (1 mg/mL) was added to a 10-mL volumetric flask containing 1 mL of H_2_O. Then, 0.3 mL of 5% NaNO_2_ was added and, after 5 min, 0.3 mL of 10% AlCl_3_ was added. At the 6th min, 2 mL of 1M NaOH were added and the total volume was made up to 10 mL with H_2_O. The solution was well mixed, and the absorbance was measured against the prepared blank at 510 nm. Total flavonoids were expressed as mg·g^−1^ of rutin equivalents (RE).

*Total tannins* (TT) Tannins were determined as described by Price et al. [33] followed with minor modification by Najda [34]. Sample (1.0 g) was mixed with 10 mL of 1% methanol/HCl solution in a dark bottle and shaking for 20 min at room temperature. Then the mixture was filtrated. The tannins in the supernatant were estimated by using 1 mL of supernatant and 5 mL of vanillin/HCl mixture (by mixing equal volumes of 2% vanillin in methanol and 8% methanol/HCl) in a test tube and kept for 20 min at room temperature. The formed color was determined at 500 nm. Catechin (CTCH) was used to prepare the standard curve. The total content of tannins is expressed in mg·g^−1^ DW.

#### 3.2.4. DPPH Radical Scavenging Assay.

The radical scavenging activity of the plant extracts against 2,2-diphenyl-1-picryl hydrazyl (DPPH•) radical was determined using a method [35] with slight modifications. Aliquots of the extracts of various concentrations (10–100 µg/mL) were prepared in methanol. One milliliter of these extract concentrations was placed in test tubes and methanol (3 mL) was added followed by 1 mM methanol solution of DPPH• (0.5 mL). A blank solution containing the same amount of methanol and DPPH• was also prepared. After 30 min incubation at room temperature, the absorbance was read against blank at 517 nm. Inhibition of free radical by DPPH• in percent (%) was calculated using following formula (Equation (1)):

% inhibition of DPPH• = {[Ab − Aa]/Ab} × 100(1)

where Ab is the absorption of the blank sample and Aa is the absorption of the extract. 

#### 3.2.5. Chemical Reagents

Organic solvents with spectroscopic purity were obtained from Merck (Darmstadt, Germany). All steviol glycosides with a purity ≥ 95% (reference standards), gallic acid (GAE) with a purity ≥ 95% (reference standard), caffeic acid (CAE) with a purity ≥ 95% (reference standard), rutin (RE) purity ≥95 % (reference standard), catechin (CTCH) purity ≥ 95% (reference standard) and 2,2-diphenyl-1-picryl hydrazyl was from Sigma–Aldrich (St. Louis, MO, USA). Folin and Ciocalteu reagent, Arnov reagent, acetic acid, HCl, luteolin and 1 N sodium hydroxide were from the company POCH (Gliwice, Poland).

### 3.3. Molecular Assays

#### 3.3.1. DNA Extraction

DNA was isolated from 10 randomly selected plants (fresh young leaves) in two replications, for every listed in Table 5 genotypes. DNA was extracted following the CTAB (Cetyl Trimethylammonium Bromide) method described by Doyle and Doyle [36]. The DNA concentration was determined using Thermo Scientific NanoDrop spectrophotometer (Wilmington, DE, USA). Test samples were diluted to a final concentration 20 ng/μL.

#### 3.3.2. RAPD Analysis. 

Ten 10-base primers selected from 40 arbitrary primers were used for PCR amplification (Table 3). DNA amplification for RAPD markers was carried out in a final volume of 15 μL containing 0.5 U of Taq DNA Polymerase (Fermentas, Vilnius, Lithuania), 0.3 μL of oligonucleotide primer (10 μM), 200 μM of dNTPs, 1X PCR Buffer with MgCl_2_, and 40 ng of genomic DNA as templates. The amplification was performed in a gradient thermal cycler (Biometra GmbH, Jena, Germany) with reaction conditions programmed as initial predenaturation at 94 °C for 4 min, followed by 44 cycles of denaturation at 94 °C for 1 min, annealing at 36 °C for 1 min, and extension at 72 °C for 2 min. A final extension was done for 7 min at 72 °C with a hold temperature of 4 °C. PCR products were electrophoresed in 1.5% agarose gels stained with ethidium bromide at constant voltage (3 V/cm of gel) until bromophenol blue/loading dye migrated to the other end of the gel. The gel was visualized on a UV-transilluminator and photographed using GeneSnap ver. 7.09 (SynGene, Frederick, MD, USA) a gel documentation system. GeneRuler 100bp DNA Ladder Plus was used to establish molecular weight of the products.

### 3.4. Chemical and DNA Data Analysis

An ANOVA (Statgraphics Centurion, Warrenton, VA, USA) was used to study the influence of the genotypes on the contents steviol glycosides, antioxidants, phenols, flavonoids and tannins. In this analysis, the homogenous groups indicate statistical differences between genotypes (α = 99%). Analysis of replicated values, standard errors (±), and least significant difference (LSD) was used to detect differences between means (*p* < 0.05) were carried out by using Tukey’s test. All data included in the analysis were performed in triplicate.

RAPD products (clearly identifiable and repetitive) were scored as present (1) or absent (0) based on the photographs. The band was assumed to be monomorphic if it was detected in all individuals explored. As a polymorphic profile considered bands detected in specified genotypes, specific was restricted to a particular individual. Resolving power of the primer was calculated using the formula: Resolving power (Rp) = ΣIb, where band informativeness is Ib = 1 − [2(0.5 − p)], p is the proportion of occurrence of bands in the genotypes out of the total number of genotypes [37]. Genetic pairwise similarities (SI-similarity index) between studied genotypes were evaluated according to Dice’s formula after Nei and Li [38]. A cluster analysis was conducted using the distance method UPGMA (Unweighted Pair-Group Method with Arithmetic Mean) in the program PAST [39]. The cluster analysis based on steviol glycosides (% DW) and polyphenols (mg/g^−1^ DW) concentration was conducted using the average linkage method available in STATISTICA 13.1. [40].

## 4. Conclusions

In summary, *Stevia rebaudiana* genotypes collected from different locations of the world showed clear variation at biochemical and genetic levels in Polish climate conditions. Genotypes from Morocco, Poland, Egypt and Nigeria can be defined as samples of higher quality compared to other genotypes analyzed in terms of the amount of steviol glycosides. Considering the rebaudioside A/stevioside ratio as a selection criterion, genotypes from Australia (genotype 9), China (genotype 1), India (genotype 3) and Pakistan (genotype 8) should be considered to be valuable in terms of suitability for obtaining new varieties. The present results indicated differential banding pattern and considerable polymorphism among all ten stevia genotypes using RAPD markers. Genotypes 6, 7, 2, 10, 1 and 3, as genetically different, could be selected for further breeding programs of stevia6. Patents

## Figures and Tables

**Table 1:** Concentration of steviol glycosides (% dry weight (DW)) in leaves of *Stevia rebaudiana* genotypes.

Genotypes	Stevioside	Rebaudioside A	Reb A/Stv Ratio	Rebaudioside C	Rebaudioside D	Total
**1**	2.72 ± 0.23 ^a1^	4.41 ± 0.02 ^c^	1.62	1.01 ± 0.02 ^d^	0.32 ± 0.04 ^c^	8.46 ^b^
**2**	7.66 ± 0.41 ^c^	3.73 ± 0.01 ^bc^	0.49	0.85 ± 0.02 ^c^	0.24 ± 0.01 ^b^	12.48 ^c^
**3**	2.68 ± 0.09 ^a^	4.33 ± 0.06 ^c^	1.61	0.98 ± 0.01 ^d^	0.32 ± 0.07 ^c^	8.31 ^b^
**4**	3.70 ± 0.13 ^b^	1.17 ± 0.08 ^a^	0.32	0.27 ± 0.03 ^a^	0.12 ± 0.01 ^a^	5.26 ^a^
**5**	3.61 ± 0.04 ^b^	1.22 ± 0.13 ^a^	0.34	0.28 ± 0.01 ^a^	0.12 ± 0.02 ^a^	5.22 ^a^
**6**	8.09 ± 0.23 ^c^	3.78 ± 0.03 ^bc^	0.47	0.86 ± 0.05 ^c^	0.25 ± 0.08 ^b^	12.97 ^c^
**7**	7.83 ± 0.20 ^c^	3.29 ± 0.09 ^b^	0.42	0.75 ± 0.06 ^b^	0.21 ± 0.05 ^b^	12.08 ^c^
**8**	2.73 ± 0.16 ^a^	4.34 ± 0.06 ^c^	1.59	0.98 ± 0.02 ^d^	0.34 ± 0.06 ^c^	8.39 ^b^
**9**	2.71 ± 0.08 ^a^	4.48 ± 0.02 ^c^	1.65	1.01 ± 0.02 ^d^	0.35 ± 0.01 ^c^	8.55 ^b^
**10**	7.15 ± 0.17 ^c^	3.34 ± 0.04 ^b^	0.47	0.76 ± 0.01 ^b^	0.17 ± 0.01 ^a^	11.42 ^c^
**Average**	**4.89**	**3.41**	**0.90**	**0.77**	**0.24**	**9.31**

^1^ Different letters a, b, c… in the same column indicate statistically significant differences (*p* < 0.05).

**Table 2:** Concentration of polyphenols (mg·g^−1^ DW) in leaves of *Stevia rebaudiana* genotypes.

Genotypes	Total Phenols	Total Flavonoids	Phenolic Acid	Total Tanins	DPPH•
**1**	22.28 ± 0.01 ^b1^	10.19 ± 0.11 ^e^	2.34 ± 0.12 ^b^	1.56 ± 0.06 ^a^	24.04 ± 0.17 ^a^
**2**	16.14 ± 0.07 ^a^	12.94 ± 0.19 ^f^	1.22 ± 0.01 ^a^	1.11 ± 0.01 ^a^	48.16 ± 0.29 ^d^
**3**	26.27 ± 0.04 ^c^	6.60 ± 0.29 ^ab^	3.24 ± 0.11 ^c^	1.51 ± 0.15 ^a^	21.05± 0.08 ^a^
**4**	26.27 ± 0.05 ^c^	6,51 ± 0.10 ^a^	3.43 ± 0.21 ^cd^	1.86 ± 0.09 ^a^	32.69 ± 0.30 ^b^
**5**	27.81 ± 0.03 ^c^	5.87 ± 0.14 ^a^	4.06 ± 0.22 ^d^	1.76 ± 0.12 ^a^	29.10 ± 0.27 ^b^
**6**	14.91 ± 0.24 ^a^	12.99 ± 0.23 ^f^	1.01 ± 0.04 ^a^	1.45 ± 0.07 ^a^	43.25 ± 0.33 ^c^
**7**	15.88 ± 0.11 ^a^	12.50 ± 0.14 ^f^	1.66 ± 0.06 ^a^	1.41 ± 0.01 ^a^	47.04 ± 0.24 ^cd^
**8**	25.25 ± 0.04 ^c^	7.32 ± 0.04 ^c^	3.09 ± 0.10 ^c^	1.75 ± 0.05 ^a^	20.58 ± 0.08 ^a^
**9**	24.61 ± 0.01 ^b^	9.18 ± 0.05 ^d^	2.72 ± 0.08 ^bc^	1.84 ± 0.10 ^a^	20.23 ± 0.11 ^a^
**10**	15.83 ± 0.09 ^a^	12.38 ± 0.16 ^f^	1.62 ± 0.14 ^a^	1.42 ± 0.03 ^a^	45.08 ± 0.25 ^cd^
**Average**	**21.52**	**9.65**	**2.44**	**1.57**	**33.12**

^1^ Different letters a, b, c… in the same column indicate statistically significant differences (*p* < 0.05).

**Table 3:** Primer sequence, number of products and other parameters for each RAPD primer.

No.	Primer Sequence 5′-3′	Number of Products						Rp ^2^
		Total	Polymorphic	Specific	Monomorphic	% P ^1^	Range of Size (bp)	
**RAPD 1**	CGATTGGACG	9	7	1	1	77.8	600–2000	4.0
**RAPD 2**	ATGCCGCGAT	10	7	0	3	70.0	400–2100	5.2
**RAPD 3**	TAGCGCCAAT	11	7	1	3	63.7	600–2800	5.0
**RAPD 4**	TTAAGGCCT	12	9	1	2	75.0	600–1600	6.4
**RAPD 5**	CACCCGATGA	3	2	0	1	66.7	400–2200	1.2
**RAPD 6**	ATGTGCCGTA	8	6	1	1	75.0	700–2200	4.6
**RAPD 7**	TGGCGCAATA	5	4	0	1	80.0	800–2200	2.2
**RAPD 8**	ACAACGCCTC	8	5	1	2	62.5	700–1000	4.4
**RAPD 9**	GACCGCTTTG	8	7	0	1	87.5	800–2200	4.8
**RAPD 10**	CCTCCTCATC	5	3	1	1	60.0	700–2200	2.0
**Average/primer**		7.9	5.7	0.6	1,6	-	-	3.98
**Total**		79	57	6	16	72.2	400–2200	-

^1^ Percentage of polymorphism; ^2^ Rp—resolving power.

**Table 4:** Genetic similarity estimated among 10 stevia genotypes, based on RAPD markers calculated by Dice’s coefficient.

GENOTYPE	1	2	3	4	5	6	7	8	9	10
**1**	1	0.56	0.86	0.86	0.87	0.58	0.59	0.64	0.50	0.57
**2**		1	0.58	0.62	0.61	0.81	0.74	0.67	0.80	0.73
**3**			1	0.82	0.80	0.56	0.54	0.67	0.52	0.57
**4**				1	0.92	0.54	0.58	0.68	0.54	0.59
**5**					1	0.59	0.59	0.64	0.55	0.60
**6**						1	0.91	0.72	0.90	0.78
**7**							1	0.76	0.83	0.70
**8**								1	0.70	0.67
**9**									1	0.81
**10**										1

**Table 5:** Seed origin.

Genotypes	Origin
**1.**	China (Pingnan Junong Mountain Farming Specialized Farmers Cooperative, Ningde, 26°40′N 119°31′E)
**2.**	Poland (Zgierz 51°51’N, 19°24′E)
**3.**	India (Sneartha Bio Tech Private Limited, Ahmedabad, 23°1′N, 72°34′E)
**4.**	Malaysia (Agricultural Research and Development Institute, Serdang, 3°20′N, 101°30′E)
**5.**	Paraguay (Plant Word Sedds, Capiatá, 25º21′S, 57º25′W)
**6.**	Morocco (Regional Centre of Agronomic Research of Tangier, 35°46′N 5°48′W)
**7.**	Egipt (Giza Seeds and Herbs, Giza, 30°00′N, 31°12′E)
**8.**	Pakistan (University of Agriculture, Faisalabad, 31°25′N, 73°5′E)
**9.**	Australia (Central Queensland University, Rockhampton, 23°22′S, 150°30′E)
**10.**	Nigeria (Herbarium of Obafemi Awolowo University, Ile-Ife, 7°28′N, 4°34′E)
